# *LINC00261* and the Adjacent Gene *FOXA2* Are Epithelial Markers and Are Suppressed during Lung Cancer Tumorigenesis and Progression

**DOI:** 10.3390/ncrna5010002

**Published:** 2018-12-28

**Authors:** Sonam Dhamija, Andrea C. Becker, Yogita Sharma, Ksenia Myacheva, Jeanette Seiler, Sven Diederichs

**Affiliations:** 1 Division of Cancer Research, Department of Thoracic Surgery, Medical Center—University of Freiburg, Faculty of Medicine, University of Freiburg, 79106 Freiburg, Germany; s.dhamija@dkfz.de (S.D.); andrea.becker@uniklinik-freiburg.de (A.C.B.); yogita.sharma@med.lu.se (Y.S.); ksenia.myacheva@uniklinik-freiburg.de (K.M.); 2 German Cancer Consortium (DKTK), Partner Site Freiburg, 79106 Freiburg, Germany; 3 Division of RNA Biology & Cancer, German Cancer Research Center (DKFZ), 69120 Heidelberg, Germany; jeanette.seiler@dkfz-heidelberg.de; 4 CellNetworks Excellence Cluster, University of Heidelberg, 69120 Heidelberg, Germany

**Keywords:** lncRNA, non-coding RNA, metastasis, EMT, lung cancer, *FOXA2*, cell migration

## Abstract

Lung cancer continues to be the leading cause of cancer-related deaths worldwide, with little improvement in patient survival rates in the past decade. Long non-coding RNAs (lncRNAs) are gaining importance as possible biomarkers with prognostic potential. By large-scale data mining, we identified *LINC00261* as a lncRNA which was significantly downregulated in lung cancer. Low expression of *LINC00261* was associated with recurrence and poor patient survival in lung adenocarcinoma. Moreover, the gene pair of *LINC00261* and its neighbor *FOXA2* were significantly co-regulated. *LINC00261* as well as *FOXA2* negatively correlated with markers for epithelial-to-mesenchymal transition (EMT) and were suppressed by the EMT inducer TGFβ. Hierarchical clustering of gene expression data from lung cancer cell lines could further verify the association of high *LINC00261*/*FOXA2* expression to an epithelial gene signature. Furthermore, higher expression of the *LINC00261*/*FOXA2* locus was associated with lung cancer cell lines with lower migratory capacity. All these data establish *LINC00261* and *FOXA2* as an epithelial-specific marker pair, downregulated during EMT and lung cancer progression, and associated with lower cell migration potential in lung cancer cells.

## 1. Introduction

Cancer is a multigenic disease and unifying principles governing the molecular mechanisms of cancer progression and metastasis are yet to be defined [1]. Large-scale gene expression profiling studies have established the molecular heterogeneity of tumors and there is a constant search for novel biomarkers with predictive value in early diagnosis, prognosis, and prediction of recurrence or progression [2,3,4,5]. The non-coding transcriptome is emerging as a major source for candidates in this direction [6]. Several non-coding RNAs (ncRNAs) including microRNAs (miRNAs) and long non-coding RNAs (lncRNAs) have been reported as predictive markers for tumorigenesis and tumor progression in diverse malignancies [7,8,9].

Metastasis, a process by which tumor cells spread from their primary site to distant locations in the body, is a characterizing feature of aggressive tumors and constitutes the major reason for cancer-related fatalities [10]. The role of cell migration and epithelial-to-mesenchymal transition (EMT) in metastatic progression is now well established [11]. An epithelial tumor needs to trans-differentiate into a mesenchymal-like phenotype to actively degrade the extra-cellular matrix and translocate through the tissue and blood stream. A reversal of this process of EMT, referred to as MET, could then be important for tumor formation at distant sites [12]. Thus, a coherent set of spatial and temporal events are required for the establishment of metastatic lesions. In addition to protein-coding genes constituting transcription factors and epigenetic regulators, miRNAs, and lncRNAs contribute to this process [13,14]. 

Lung cancer is the most common form of cancer world-wide. While there has been a steady increase in survival for most cancers, the five-year survival for lung cancer is currently only 18% [15]. As the name suggests, the lncRNA metastasis associated in lung adenocarcinoma transcript 1 (MALAT1), one of the earliest lncRNA linked to cancer, is a biomarker for metastasis in lung cancer [16]; *MALAT1* is not only a marker, but also an essential factor and potential therapeutic target in lung cancer metastasis [8]. Here, we utilized a similar approach to analyze the non-coding transcriptome of lung adenocarcinoma (LUAD) and lung squamous cell carcinoma (LUSC) tumor samples for genes associated with progression and identified *LINC00261* and its neighboring gene *FOXA2* as probable tumor suppressors with strong association to non-migrating epithelial cells.

## 2. Results

### 2.1. Low Expression of LINC00261 Correlates with Metastatic Progression and Predicts Poor Survival in LUAD

In search of lncRNAs relevant to lung cancer progression and metastasis, we performed large-scale data mining of the RNA-Seq data from primary LUAD obtained from The Cancer Genome Atlas (TCGA, Bethesda, MD, USA) via the TANRIC database [17]. For an in-depth analysis, the patient details available at the TCGA data portal were filtered on the basis of the clinical outcome of disease: patients were selected that either developed a metastasis or recurrence within the first 24 months after R0 surgery or that were disease-free for at least 24 months after surgery. Ninety-eight of the 488 RNA-Seq samples could be matched with these clinical evaluation parameters to be classified as disease-free post-surgical intervention or recurring/progressing to metastasis (Table 1).

**Figure 1:**
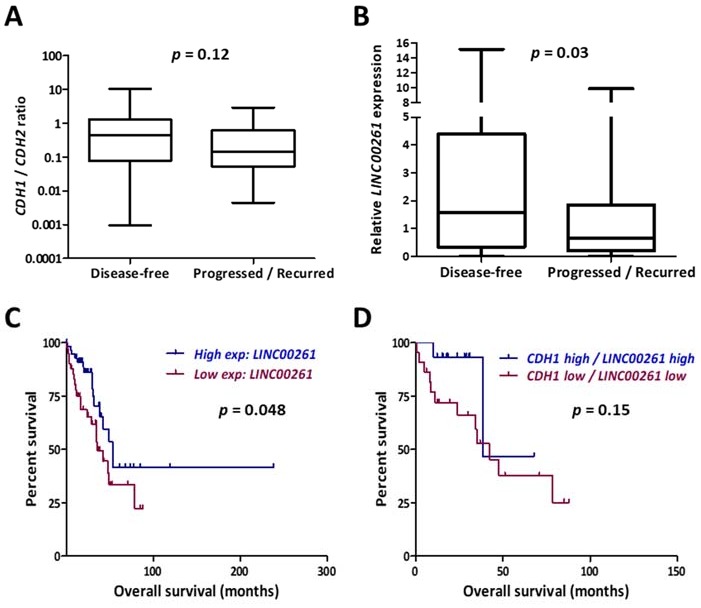
LINC00261 is a biomarker for progression and poor survival in lung adenocarcinoma. (**A**) Ratio of gene expression values for *CDH1* and *CDH2* were calculated for the 98 LUAD tumor samples described and categorized into disease-free (*n* = 44) and progressed/recurred (*n* = 54) in Table 1. Low ratios in progressed/recurred samples indicate positive correlation with invasive cells. (**B**) *LINC00261* expression values from the same dataset showing significantly lower levels of expression in progressed/recurred samples. (**C**,**D**) Kaplan–Meier plot showing the association of *LINC00261* expression (**C**) or *LINC00261/CDH1* expression (**D**) with patient survival in LUAD (all stages). *p-*value refers to log-rank *p*-value.

The RNA-Seq data from these two cohorts possessed comparable patient parameters except for the clinical outcome in cancer progression and were compared to identify novel prognostic markers for lung cancer progression. A high E-cadherin (*CDH1*)/N-cadherin (*CDH2*) ratio is considered as an epithelial signature which would suggest low metastatic potential. As expected, the disease-free group displayed higher *CDH1*/*CDH2* ratios, even though the values were not statistically significant (Figure 1A). In contrast, the lncRNA *LINC00261* emerged as a novel candidate which showed significant downregulation in primary LUAD tumors which recurred or gave rise to metastatic lesions at distant sites (Figure 1B). Consistently, low expression of *LINC00261* was significantly associated with poor patient survival in LUAD (Figure 1C). We analyzed whether an EMT-related signature with prognostic value could be developed by combined monitoring of *LINC00261* and *CDH1* expression. However, low *CDH1/LINC00261* expression signature did not reveal significant association with patient survival (Figure 1D).

### 2.2. LINC00261 Expression is Downregulated in Lung Cancer

**Figure 2:**
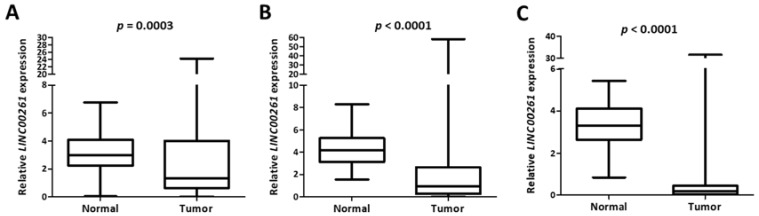
*LINC00261* is suppressed in lung cancer. (**A**–**C**) The *LINC00261* expression data from LUAD-Korea (*n* = 77 normal/83 tumor). (**A**) LUAD-TCGA (*n* = 55 normal/486 tumor) and (**B**) lung squamous cell carcinoma (LUSC)-TCGA (*n* = 17 normal/220 tumor). (**C**) Datasets are represented as box plots.

To establish whether the expression of *LINC00261* was generally decreased between normal tissues and primary lung tumor samples, we employed two independent LUAD cohorts with publicly available gene expression data obtained by two independent, but complementary methods: RNA-Seq data from TCGA (“TCGA”) or microarray data from an independent cohort (“Korea”) [18]. Interestingly, we observed a strong and significant suppression of *LINC00261* in both LUAD cohorts compared to the respective normal tissue controls (Figure 2A,B). The same was observed for lung squamous cell carcinoma in the TCGA LUSC cohort (Figure 2C). This was suggestive of a general tumor suppressor function for this lncRNA in lung cancer.

### 2.3. LINC00261 Expression Strongly Correlates with the Neighboring FOXA2 Gene

**Figure 3:**
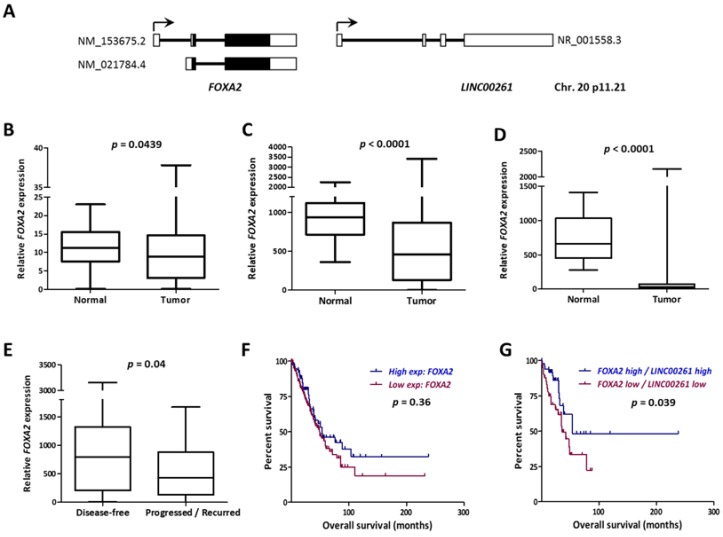
*FOXA2* gene neighboring *LINC00261* is co-downregulated in lung cancer. (**A**) The *FOXA2*/*LINC00261* gene locus on the small arm of human chromosome 20. Black boxes indicate coding exons and white boxes indicate non-coding exons. The accession numbers for reference transcripts are indicated. (**B**–**D**) The *FOXA2* expression data from LUAD-Korea (*n* = 77 normal/83 tumor), (**B**) LUAD-TCGA (*n* = 55 normal/486 tumor), and (**C**) the LUSC-TCGA (*n* = 17 normal/220 tumor). (**D**) Datasets are represented as box plots. (**E**) *FOXA2* expression is significantly low in progressed/recurred LUAD tumor samples described in Table 1. (**F**,**G**) Kaplan–Meier Plot showing the association of *FOXA2* expression or (**F**) *LINC00261/FOXA2* expression with (**G**) patient survival in LUAD (all stages). *p*-value refers to log-rank *p*-value.

The *LINC00261* gene and its genomic context are highly conserved in human and mouse genomes and it is placed closely downstream to the *FOXA2* gene in sense direction (Figure 3A). *FOXA2* is an established negative regulator of metastasis [19]. Notably, we found *FOXA2* also significantly downregulated in LUAD and LUSC tumors in all three cohorts (Figure 3B–D). More importantly, similar to *LINC00261*, low expression of *FOXA2* was also indicative of higher recurrence in LUAD (Figure 3E). When the lncRNA and messenger RNA (mRNA) data from LUAD were merged and compared, *FOXA2* showed the strongest correlation with *LINC00261* expression in both normal and tumor samples independently in the LUAD and the LUSC datasets (Figure S1). Since low *LINC00261* expression was associated with poor patient survival in LUAD, we analyzed whether the same was true for *FOXA2*. Even though Kaplan–Meier survival analysis did not reveal significant differences in survival between *FOXA2* low- and high-expression groups, a low *FOXA2/LINC00261* co-expression signature was strongly associated with poor patient survival (Figure 3F,G).

### 2.4. LINC00261/FOXA2 Expression is Indicative of an Epithelial Gene Signature in Lung Cancer and is Suppressed in TGFβ-Induced EMT

**Figure 4:**
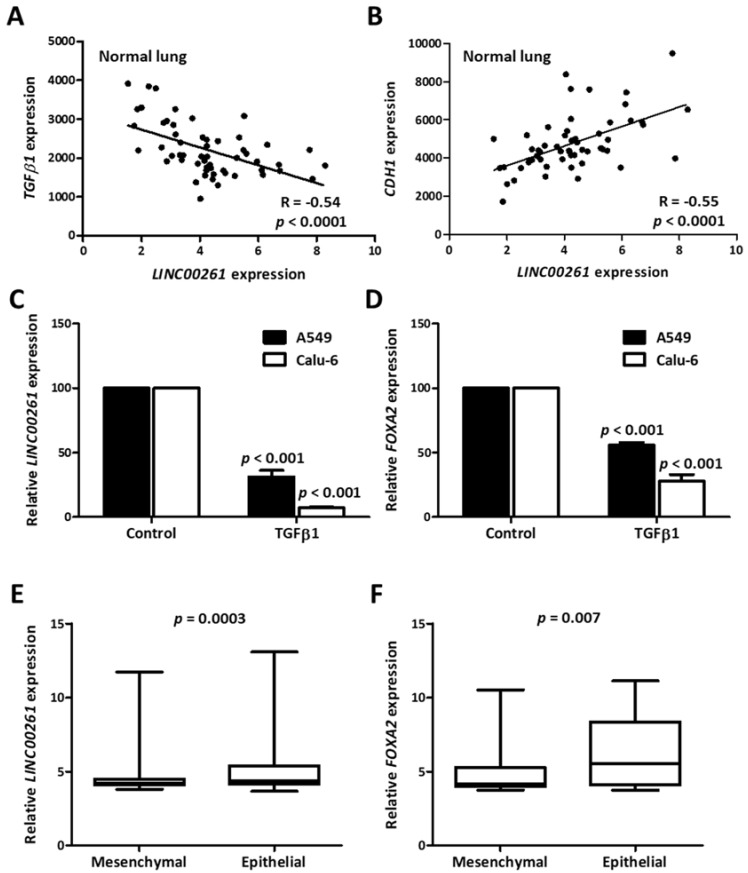
*LINC00261* and *FOXA2* are indicative of an epithelial gene signature in normal lung and lung cancer cell lines. (**A**,**B**) Correlation plots showing strong correlation between *LINC00261* and *TGFβ1* (**A**)/*CDH1*. (**B**) Expression in normal lung samples. (**C**,**D**) *LINC00261* (**C**) and *FOXA2* (**D**) are downregulated during TGFβ-induced epithelial-to-mesenchymal transition (EMT) in A549 and Calu-6 lung cancer cell lines (*n* = 3). *p*-value was calculated using ANOVA test. (**E**,**F**) The expression of *LINC00261* (**E**) and *FOXA2* (**F**) is stronger in the epithelial cluster of lung cancer cell lines generated by hierarchical clustering based on a previously established EMT signature (*n* = 62 mesenchymal/123 epithelial cell lines).

To understand the contribution of *LINC00261* towards cancer progression and metastasis, we monitored the correlation of *LINC00261* expression with that of *TGFβ1* and the epithelial marker *CDH1*. In the non-cancerous control samples with high *LINC00261* expression, we observed a strong negative and positive correlation with *TGFβ1* and *CDH1* expression, respectively (Figure 4A,B). A negative association with the EMT-inducer *TGFβ1* and the strong positive correlation with *CDH1* was indicative of epithelial-specific expression of *LINC00261*. To verify this hypothesis, we treated two lung cancer cell lines, which displayed high endogenous *LINC00261* expression, with TGFβ1 to induce EMT and quantified *LINC00261* expression. In both cell lines, there was a strong TGFβ-dependent suppression of *LINC00261* expression, establishing that *LINC00261* was an epithelial marker (Figure 4C). Consistent with the co-expression of *LINC00261/FOXA2* in lung cancer samples, TGFβ1 also induced a significant suppression of *FOXA2* (Figure 4D). To monitor whether this association of the *LINC00261*/*FOXA2* locus with epithelial cells and epithelial markers was a broadly conserved process in lung cancer, we further employed a sophisticated bioinformatic analysis. A recent study identified a 76-gene EMT signature to categorize NSCLC cell lines into epithelial and mesenchymal subgroups [20]. We utilized this gene signature and performed hierarchical clustering analysis with a large-scale lung cancer cell line gene expression dataset from the Cancer Cell Line Encyclopedia (CCLE) [21] revealing two distinct clusters (Figure S2), which showed a strong intra-group correlation. Analysis of EMT markers *CDH1* and *VIM* in the clusters could conclusively verify the identity of cluster 1 as mesenchymal and cluster 2 as epithelial (Figure S3). When *LINC00261* and *FOXA2* expression was monitored in these two clusters, a significant upregulation of these genes was observed in the epithelial cluster (Figure 4E,F), once again verifying the association of the *LINC00261*/*FOXA2* locus with epithelial cell lineages.

### 2.5. LINC00261 Expression Negatively Correlates with Migration Capacity in Lung Cancer Cell Lines

**Figure 5:**
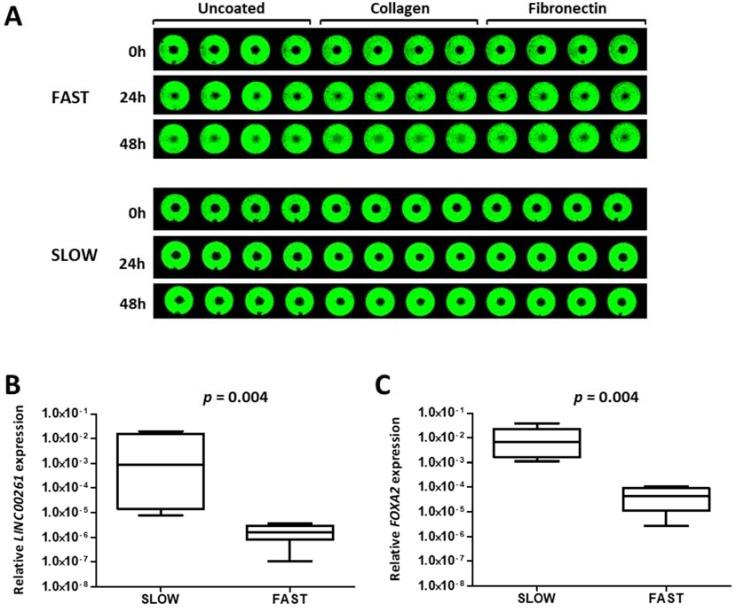
*LINC00261* is a negative regulator of lung cancer cell migration. (**A**) Representative pictures showing a slow (NCI-H1993) and a fast (NCI-H2030) migrating cell line in the cell migration assay with the Oris system. Near infrared-fluorescent scans of cell monolayer are shown with the average percentage of the central free area for migration at 0, 24 h, and 48 h. (**B**,**C**) *LINC00261* (**B**) and *FOXA2* (**C**) expression in fast (*n* = 5) and slow (*n* = 6) migrating cell lines showing high expression of both genes in slow migrating cells.

The comparative analysis of tumor samples clearly established *LINC00261* as a biomarker with consistent reduction in tumors with propensity to progress towards metastasis. In addition, clustering analysis and the downregulation during TGFβ1-induced EMT defined an association with non-mesenchymal cells. Hence, we explored whether *LINC00261* could be associated with cell migration which is linked to lung cancer progression. We employed our data for a panel of lung cancer cell lines which were analyzed for their capacity to migrate on a cell culture-treated surfaces or surfaces coated with the extracellular matrix proteins collagen or fibronectin [22]. After classifying the cell lines into distinct sets of fast and slow migrating cell lines (Figure 5A), we analyzed the expression of *LINC00261* and *FOXA2* in these two groups of cell lines. Importantly and consistent with the expression pattern in the primary tumor samples, both genes derived from the *LINC00261*/*FOXA2* locus were strongly downregulated in the fast migrating lung cancer cell lines compared to the slow migrating cell lines (Figure 5B,C).

## 3. Discussion

Cancer progression is a multistep process associated with stage-specific alterations in the transcriptome leading to uncontrolled proliferation, resistance to cell death, and increased invasiveness. Several lncRNA transcripts have been shown to display cancer-specific expression patterns, making them potential candidates as biomarkers and targets in cancer diagnosis and therapy. Comparison of gene expression between tumor and normal samples has been the most common approach to identify potential cancer gene candidates—for mRNAs as well as lncRNAs. The prototype candidate is the nuclear lncRNA MALAT1 which was originally identified as a metastasis-associated transcript in lung cancer. Further studies have established a functional role for *MALAT1* in metastatic progression, with participation in splicing and transcriptional regulation [8]. Here, we have made use of publicly available tumor transcriptome datasets for lung adenocarcinoma and lung squamous cell carcinoma and compared it to the clinical stratification information to identify lncRNAs associated with lung cancer progression and recurrence. The top candidate identified by our analysis was *LINC00261* which was strongly downregulated in lung cancer. Patients with primary LUAD tumors with low expression of *LINC00261* were more prone to develop metastatic lesions indicating a potential prognostic value of this transcript. Interestingly, several genome-wide studies verified this cancer-specific expression profile of *LINC00261* and a recent study also reported low *LINC00261* expression as a prognostic marker for non-small cell lung cancer corroborating our results [23,24,25,26].

*LINC00261* was originally identified and reported as definitive endoderm-associated lncRNA1 (*DEANR1*). In the non-tumor settings, *LINC00261* is strongly expressed in endodermal tissues and drives the endoderm differentiation by upregulating the expression of the endoderm differentiation factor FOXA2. Interestingly, we observed a positive correlation between the expression of *LINC00261* and its neighboring gene *FOXA2* in normal as well as lung tumor samples, with both genes being suppressed during tumor progression as well as TGFβ-induced EMT. There are some parallels between tumor progression and early stages of endoderm differentiation, where *LINC00261*/*DEANR1* acts in *cis* by recruiting the EMT-associated transcription factors SMAD2/3 to the *FOXA2* promoter. While we could see that *LINC00261* expression is *FOXA2-*dependent, the inverse was not true in the lung cancer cell lines tested (Figure S4). This could indicate a difference in the transcriptional network between endoderm and lung cancer cell lines. However, it should be noted that the *LINC00261* knockdown efficiencies achieved here were moderate and the residual lncRNA expression could be sufficient to support *FOXA2* expression. FOXA2 is an established epithelium-specific transcription factor with a role in normal epithelial differentiation [8,27,28]. Consequently, it suppresses lung and breast cancer progression and metastasis by opposing EMT [19,29]. Our study showed that *LINC00261* is co-regulated with its neighbor *FOXA2*, and thus could play a role in FOXA2-mediated epithelial-specific transcription program. In addition to the normal versus tumor comparisons and co-regulation during TGFβ-induced EMT in representative lung cancer cell lines, we could further expand our analysis to a large panel of 185 lung cancer cell lines. By using a recently developed gene signature matrix for hierarchical clustering, we could categorize these cell lines into epithelial and mesenchymal subtypes and *LINC00261/FOXA2* expression was indeed strongly upregulated in the epithelial cluster. More importantly, when the expression of *LINC00261/FOXA2* transcripts were correlated with the experimentally verified group of fast and slow migrating lung cancer cell lines, higher expression was evidently associated with the slow migrating cell line panel. All these data indicated that there is an inverse correlation between *LINC00261*/*FOXA2* expression and EMT and cell motility.

Whether *LINC00261* plays any functional role in lung cancer progression or EMT is an unanswered question, but its overexpression in gastric cancer suppressed metastasis by direct interaction and degradation of the EMT-associated transcription factor Slug [30]. Overexpression of *LINC00261* also suppresses colon cancer metastasis by sequestering β-catenin in the cytoplasm, facilitating its degradation and suppressing Wnt signaling. Moreover, low *LINC00261* expression also correlates with cisplatin resistance in colon cancer cell lines and overexpression sensitizes them to anticancer DNA-damaging agents [31]. Thus, *LINC00261* is a lncRNA associated with diverse forms of cancer, is highly conserved evolutionarily, and is co-regulated with its neighboring gene *FOXA2* during EMT and tumorigenesis. The *LINC00261/FOXA2* expression signature presented here seems to be of great potential in predicting the disease aggressiveness and progression of LUAD. In addition to the prognostic value, suppression of the *LINC00261/FOXA2* locus may be a targetable node in the process of pathological EMT during cancer progression. Our data provides an important basis for future investigations on the role of the *LINC00261/FOXA2* axis in lung cancer tumorigenesis, progression and metastasis to characterize their tumor suppressive and anti-metastatic functions in lung cancer.

## 4. Materials and Methods

### 4.1. Cell Culture, Reagents and Treatments

All cell lines were obtained from the American Type Culture Collection (ATCC, Manassas, VA, USA) and were periodically authenticated using Multiplex Cell Authentication by Multiplexion (Heidelberg, Germany). A549 cells were cultured in Dulbecco’s Modified Eagles Medium (DMEM, SIGMA, St. Louis, MO, USA, D5671) supplemented with 10% fetal bovine serum (FBS) and 2 mM L-Glutamine. All other cell lines were propagated in RPMI 1640 medium (Thermo Scientific, Waltham, MA, USA A1049101) supplemented with 10% FBS. Wherever indicated, cells were treated with 5 ng/mL TGFβ1 (Peprotech, Rocky Hill, NJ, USA, 100-21B) for 24 h and processed for RNA isolation and RT-qPCR. 

### 4.2. RNA Isolation, cDNA Synthesis and RT-qPCR

RNA was isolated using TRI reagent (SIGMA, St. Louis, MO, USA T9424) according to the manufacturer’s protocol. RNA samples were treated with Turbo DNase (Thermo Scientific, Waltham, MA, USA AM2238) and re-purified using Phenol:Chloroform:Isoamyl alcohol (25:24:1) (Carl-Roth, Karlsruhe, Germany X985). Reverse transcription was carried out with 1 µg RNA using random-hexamer primers with the Revertaid Reverse transcriptase (ThermoFisher, Waltham, MA, USA EP0442). The 1:40 diluted cDNAs were analyzed by SYBR Green RT-qPCR (Power SYBR^TM^ Green PCR Master Mix, ThermoFisher, Waltham, MA, USA 4367659) using the Applied Biosystems StepOne plus thermal cycler. Relative transcript expression was calculated by the comparative Ct-method (2^-ddCt^). GAPDH was used as reference gene. RT-qPCR primers used in the study include: *LINC00261*- sense ACATTTGGTAGCCCGTGGAG, antisense TCTTCCCCGGAGAACTAGCA; *FOXA2-* sense GAGACTTTGGGGAGACGGTG, antisense CGGGTGAAGAAGACTGCTGT; GAPDH- sense GTGAAGGTCGGAGTCAACG, antisense TGAGGTCAATGAAGGGGTC.

### 4.3. Analysis of Cell Migration

Lung cancer cell lines were stained with the lipophilic tracer DiR (Biotium, Fremont, CA, USA 60017) and seeded as described previously [32]. Cell migration was imaged in tricoated 96-well ORIS cell migration plates (Platypus Technologies, Madison, WI, USA) using the Li-COR Odyssey infra-red scanner.

### 4.4. Knockdown Experiments

For *LINC00261* and *FOXA2* knockdown, NCI-H520 and Calu-6 cell were seeded in 6-well plates at a density of 5 × 10^5^ and 2 × 10^5^ cells per well, respectively. On day 2, cells were transfected with 10 nM of control siPOOL, siPOOL for *FOXA2* and two siPOOLs for *LINC00261* (siTOOLs Biotech, Planegg, Germany) using Lipofectamine RNAiMAX reagent (Thermo Scientific, Waltham, MA, USA 13778150). Seventy-two hours post transfection, cells were lysed in 1 mL TRI reagent (Sigma, St. Louis, MO, USA) for RNA extraction and further analysis by RT–qPCR. siPOOLs sequences are listed in Table S1.

### 4.5. Data Analysis and Statistics

Long ncRNA expression data in LUAD and LUSC were downloaded from TANRIC (https://bioinformatics.mdanderson.org/main/TANRIC:Overview) and the respective clinical data for the TCGA-LUAD dataset was obtained from the cBIO portal (http://www.cbioportal.org/index.do). The filtering parameters and the final stage-specific analysis is presented in the text and in Table 1. For correlation studies, the expression data for coding genes (*FOXA2, TGFB1*, and *CDH1*) in lung cancer samples were also obtained from the cBIO portal. Clinical data with matched gene expression z-scores for 508 LUAD samples were downloaded from cBIO portal, classified as high (>+0.5 z-score) or low (<−0.5 z-score) expression and were used for generating Kaplan–Meier Plots using GraphPad Prism 5. 

For the hierarchical clustering analysis, a microarray expression dataset for 185 lung cancer cell lines was obtained from CCLE (https://portals.broadinstitute.org/ccle). Using R, a (Pearson) correlation matrix was computed from the gene expression dataset by applying a 76-gene epithelial/mesenchymal signature reported earlier [20]. Since nine genes from the signature were not represented in the microarray datasets, these were excluded from the analysis. Hierarchical clustering was performed using standard R heatmap function (distance euclidian, method complete). The two distinct clusters were verified to be epithelial and mesenchymal by monitoring *CDH1* and *VIM* expression in the dataset.

All data were processed using the Microsoft Excel 2010 program (Microsoft, Redmond, WA, USA). Graphs were plotted and statistical analysis was performed using GraphPad Prism 5. Statistical significance was assessed using Mann–Whitney tests unless stated otherwise in the respective figure legends. The *p*-values are displayed in respective figure panels.

## Financial Disclosure Statement

S.D. (Sven Diederichs) is co-owner of siTOOLs Biotech GmbH, Martinsried, Germany.

## Figures and Tables

**Table 1:** Clinical parameters for the 98 lung adenocarcinoma (LUAD) from The Cancer Genome Atlas (TCGA) samples categorized based on the clinical outcome of tumor progression.

Parameters	Disease-Free	Recurred/Progressed
Patients	44	54
N0	27	31
N1	11	16
N2	5	7
NX	1	0
Stage IA	12	9
Stage IB	14	17
Stage IIA	3	6
Stage IIB	10	14
Stage IIIA	5	8
Median Age (years)	67.0	67.0
Age Range (years)	41–83	42–84
Male	18	25
Female	26	29
Neoadjuvant Treatment	0	0
Smoking History	37	47
Lifelong Non-Smoker	6	7

## Supplementary Materials

The following are available online at http://www.mdpi.com/2311-553X/5/1/2/s1, Figure S1. Positive correlation of *LINC00261* and *FOXA2* in lung cancer. Figure S2. Heat map showing the results of hierarchical clustering of gene expression data from lung cancer cell lines. Figure S3. E-cadherin (*CDH1*) and Vimentin (*VIM*) expression in lung cancer cell line clusters. Figure S4. *FOXA2* regulates *LINC00261* expression while *LINC00261* knockdown does not affect *FOXA2* expression. Table S1. Sequences of siPOOL used as negative control and siPOOLs targeting *FOXA2* and *LINC00261*.
