# Supplementary material for: LINC00261 and the Adjacent Gene FOXA2 Are Epithelial Markers and Are Suppressed during Lung Cancer Tumorigenesis and Progression
Source: Noncoding RNA. 2018 Dec 28;5(1):2. doi: 10.3390/ncrna5010002 (PMC6468413; doi:10.3390/ncrna5010002)
Supplement: Supplementary file 1 [file ncrna-05-00002-s001.zip › ncrna-401971-Supplementary.docx]

Supplementary Materials

*LINC00261* and the adjacent gene *FOXA2* are epithelial markers and are suppressed during lung cancer tumorigenesis and progression

Sonam Dhamija, Andrea C. Becker, Yogita Sharma, Ksenia Myacheva, Jeanette Seiler and Sven Diederichs *


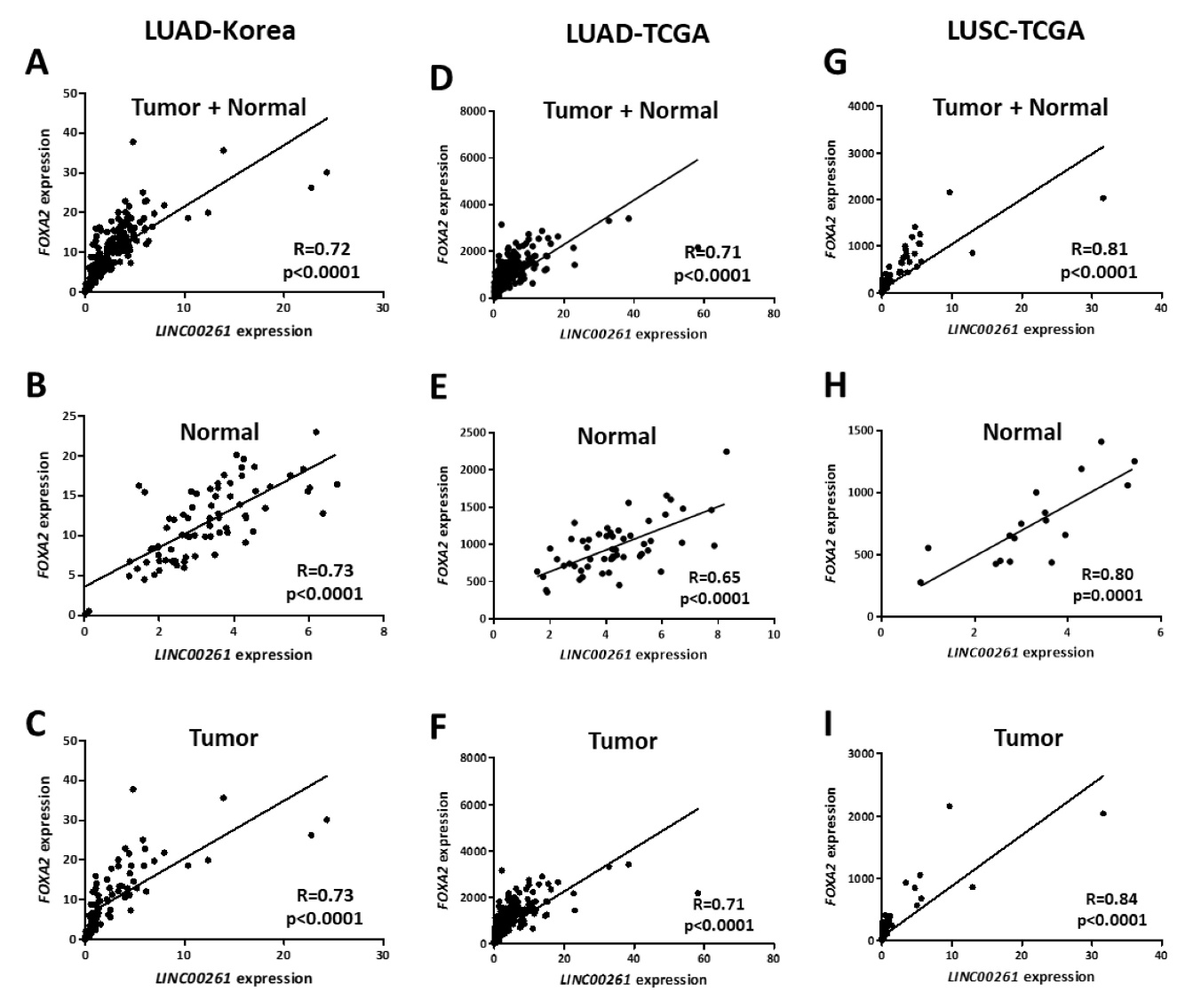


**Figure S1. Positive correlation of *LINC00261* and *FOXA2* in lung cancer. A–F.** The LUAD-Korea (**A**–**C**) and LUAD-TCGA (**D**–**F**) datasets were analyzed for correlation between *LINC00261* and *FOXA2* expression in the combined samples (**A**,**D**), normal controls (**B**,**E**) or tumor samples alone (**C**,**F**). For LUAD-Korea, 77 normal and 83 tumor samples were analyzed and the LUAD-TCGA dataset included 55 normal controls and 486 tumor samples. **G**–**I.** Similar analysis of the LUSC-TCGA dataset showing the correlation between *LINC00261* and *FOXA2* (n= 17 normal / 220 tumor). Pearson correlation coefficients (R) and p-values generated by GraphPad prism were indicated.


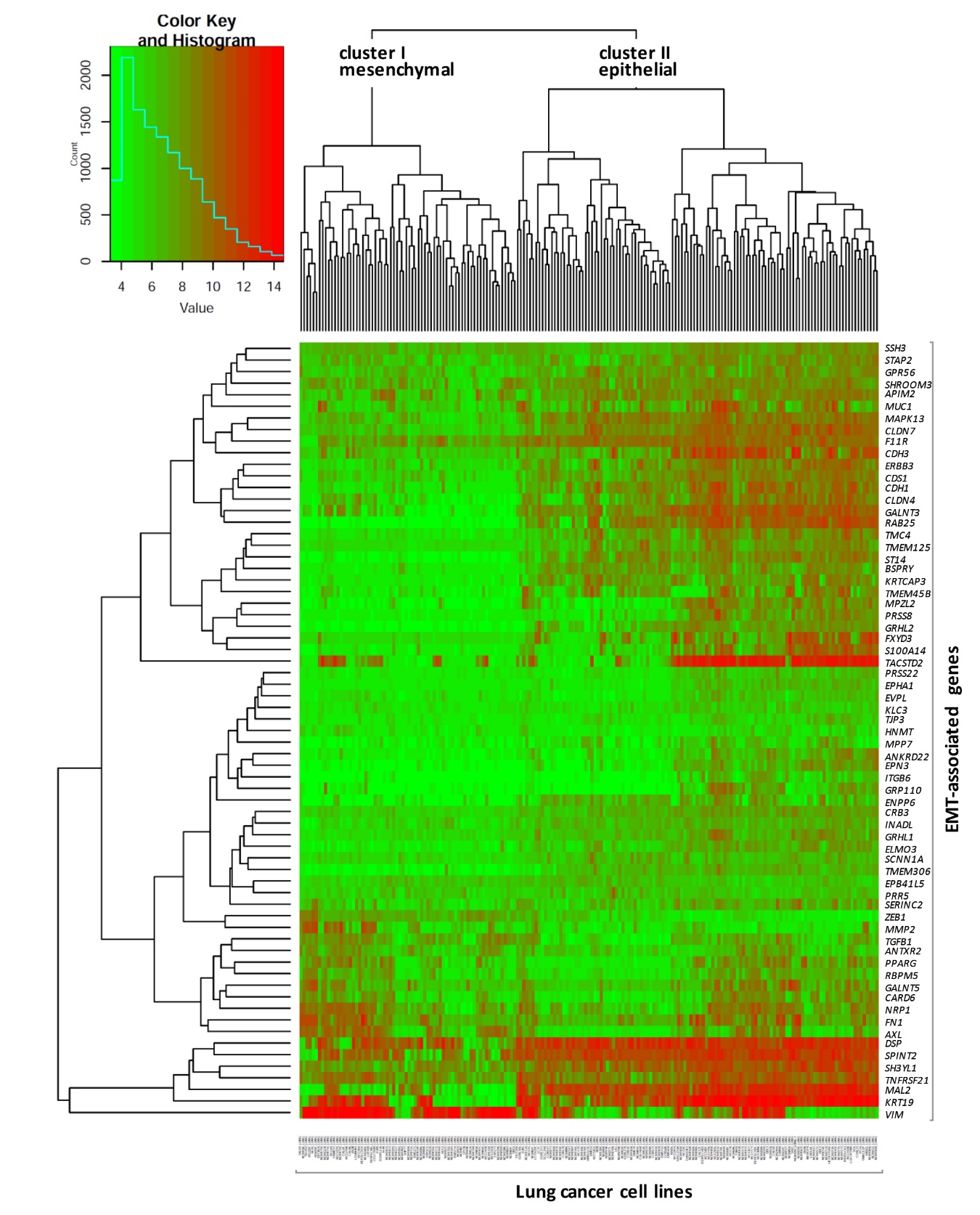


**Figure S2. Heat map showing the results of hierarchical clustering of gene expression data from lung cancer cell lines.** A 67 gene EMT signature modified from Byers et al., 2013 was used to analyse the lung cancer cell line dataset from CCLE consisting of 185 human cell lines. The analysis indicates 2 distinct clusters: mesenchymal (cluster I) and epithelial (cluster II).


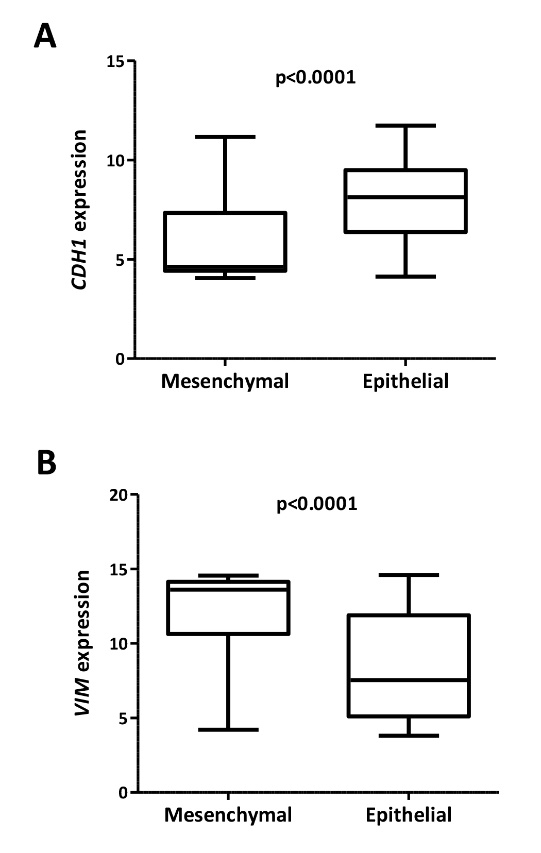


**Figure S3. E-cadherin (*CDH1*) and Vimentin (*VIM*) expression in lung cancer cell line clusters.** Expression of the epithelial marker gene *CDH1* (**A**) and the mesenchymal marker gene *VIM* (**B**) in the epithelial and mesenchymal clusters confirm their identity.


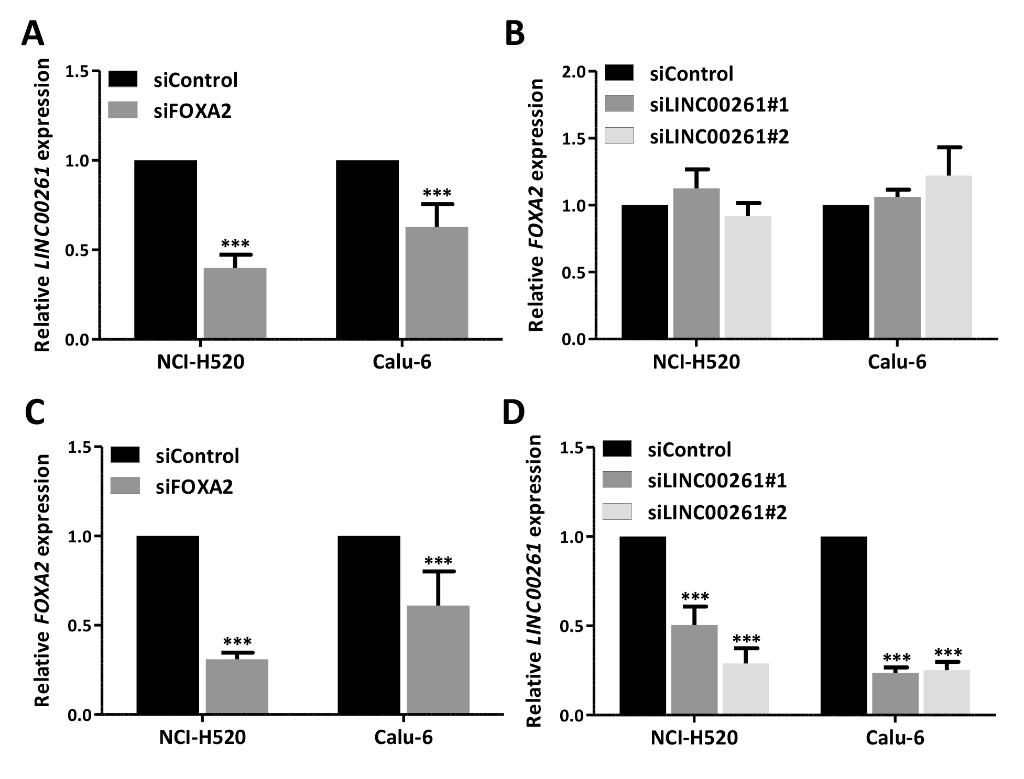


**Figure S4. *FOXA2* regulates *LINC00261* expression while *LINC00261* knockdown does not affect *FOXA2* expression.** Relative expression of *LINC00261* (**A**) and *FOXA2* (**B**) in NCI-H520 and Calu-6 cells transfected with siPOOLs targeting *FOXA2* and two different siPOOLs against *LINC00261* respectively. *FOXA2* (**C**) and *LINC00261* (**D**) were quantified in the respective knockdown samples to obtain the knockdown efficiency.

**Table S1.** Sequences of siPOOL used as negative control and siPOOLs targeting *FOXA2* and *LINC00261*.

| # | **siControl** Sequence (5’-3’) | # | **siFOXA2** Sequence (5’-3’) | # | **siLINC00261#1** Sequence (5’-3’) | # | **siLINC00261#2** Sequence (5’-3’) |
| --- | --- | --- | --- | --- | --- | --- | --- |
| 1 | TGTACGCGTCTCGCGATTT | 1 | CCGGATCGAGGACAAGTGA | 1 | GCCGCCAGAGAAGAGAGCA | 1 | GGGAGAGAAATGGACTTCA |
| 2 | TATACGCGGTACGATCGTT | 2 | CCCGGTTTTATCCCTTGAA | 2 | GGAAGGAGGTCTGTGAACA | 2 | GGCTGAAAGCTGTAGCCAT |
| 3 | TTCGCGTAATAGCGATCGT | 3 | GTGTAGACTCCTGCTTCTT | 3 | GCAGATTCAGTGACACATT | 3 | GCTGCAGCATTGCAGATAA |
| 4 | TCGGCGTAGTTTCGACGAT | 4 | CCGCAGATACCTCCTACTA | 4 | CTGAGTCAGGACAAAGATA | 4 | GGCATCCCTGCAGGGAAAT |
| 5 | TCGCGTAAGGTTCGCGTAT | 5 | GCCTCCGGTTTCCACTACT | 5 | CCTAGTCTTATATCAGTAT | 5 | CCATAGATTACACCTATAA |
| 6 | TCGCGATTTTAGCGCGTAT | 6 | CTCCTCCATTGCTGTTGTT | 6 | GGGCATCCCTGCAGGGAAA | 6 | GGGACATACAAGGTACTTT |
| 7 | TCGCGTATATACGCTACGT | 7 | GCAGCCGTTCCGTCCCAAA | 7 | GGTTGCTGGACTCAGGGTA | 7 | GCTAATTGTATTGGCTAAA |
| 8 | TTTCGCGAACGCGCGTAAT | 8 | GGACCTCAAGGCCTACGAA | 8 | GGGTACAAAGCTCCTGAAA | 8 | CCAGTAGAGCTGTTAGTTT |
| 9 | TCGTATCGTATCGTACCGT | 9 | CCCAATCTTGACACGGTGA | 9 | GCCAGGCTTCAATGTTTAA | 9 | CGCTGCCATGGTAGATATT |
| 10 | TTATCGCGCGTTATCGCGT | 10 | GGGCCAGAGTTCCACAAAT | 10 | GGCAGAGTGGGTGTACTTT | 10 | GGGCAGAGTGGGTGTACTT |
| 11 | TCTCGTAGGTACGCGATCT | 11 | CGGTGTTGCAGAGACGCAA | 11 | GCCTTGTGGGCCTCTCTTT | 11 | GCAGGAATCCCTGGATAAA |
| 12 | TCGTACTCGATAGCGCAAT | 12 | GTCTGTTGTAAATGACCAA | 12 | CCCTACGATGCAGATTTGA | 12 | GCCTCTCTTTGCTATCTAT |
| 13 | TTTGCGATACCGTAACGCT | 13 | CGGAACACCACTACGCCTT | 13 | GGGTGTGTGTTCCGAATCA | 13 | GGCAATGGTCCCAGTCCAA |
| 14 | TGCGTAAGGCATGTCGTAT | 14 | GGGCCCGGTCACGAACAAA | 14 | GGCTAAATGAGCTTTCACA | 14 | GCTTGGTTTGAGCTCAAAT |
| 15 | TTATCGGCAGTTCGCCGTT | 15 | CCCACGTTCTATATAAGGA | 15 | CTCCAGCTTAAAGCAATTA | 15 | GGAGCACAGCACCAGATTT |
| 16 | TAGCGCGACATCTATCGCT | 16 | GCTTCAGGCCCGGCTAACT | 16 | GCGTCCACTGTCCCTTCTT | 16 | GGGTTCATTGATGTGTATT |
| 17 | TCGTCGTATCAGCGCGTTT | 17 | GGAAAACGGGAAAGAATAT | 17 | GGGAAATCATATCCGACAT | 17 | GACTTAAGACTGCCTCTAA |
| 18 | TACGCGAAACTGCGTTCGT | 18 | CCCAAGACAGCAGTCTTCT | 18 | GCCACTTAGCAAGACATTT | 18 | GGATATGCAAAGCGCATTT |
| 19 | TCGACGATAGCTATCGCGT | 19 | CCATTATGAACTCCTCTTA | 19 | GCAATGGTCCCAGTCCAAA | 19 | CCCTTGAAGTCCTCAGCTT |
| 20 | TCGCGTAATACGCGATCGT | 20 | GACGCAAGGGAGAAGAAAT | 20 | CCCAACCTGTTTAAAGTAA | 20 | CAGTCACATTAAGTTGATA |
| 21 | TCGCGATAATGTTACGCGT | 21 | GCAGGGAAGTCTTACTTAA | 21 | CTCTTTGCTATCTATACAA | 21 | GGCCACTTAGCAAGACATT |
| 22 | TTAACGCGCTACGCGTATT | 22 | GCACCTGCAGATTCTGATT | 22 | GGCTTCCCAGGGCGGTTAA | 22 | GCAGGTTTCTCTGTGGAAA |
| 23 | TCGCGTATAGGTAACGCGT | 23 | GTGCTTTATTTATGGCTTA | 23 | GTCATCAACAAATAATCAA | 23 | GCATCTGCTGGAGAAGAAA |
| 24 | TTACGCGATCACGTAACGT | 24 | GTGTACTCCCGGCCCATTA | 24 | CGCTTGGTTTGAGCTCAAA | 24 | GCTCCAGCTTAAAGCAATT |
| 25 | TTATCGCGCGTCGCGTAAT | 25 | CAGGTCTCGGGTCCGATTA | 25 | GCCATTCAAGGCATCATTT | 25 | GGAACCCAACCTGTTTAAA |
| 26 | TTACGTACTAGTGCGTACT | 26 | GTGAGTGACTCGGTGTAAA | 26 | GGCCTCTGGGCAGAGACTA | 26 | CTCTGTAGAGTGGCTTTAA |
| 27 | TATACGCCGGTTGCGTAGT | 27 | CACAAATCTATATTAAAGT | 27 | GCAATCTTTGTGGGACATT | 27 | CCTGTGACATAGGTGGATA |
| 28 | TTCGCGTGCATAGCGTAAT | 28 | CCCACCTGAAGCCGGAACA | 28 | GGAAAGGCCGTGAAGCTAA | 28 | CCCACCTGTGGGAAATACA |
| 29 | TACGCGACCTAATCGCGAT | 29 | CCAAACAGAGGGCCACACA | 29 | GCCATAGATTACACCTATA | 29 | GCACACAGGGCTTCTATAA |
| 30 | TCGTACGCTGAACGCGTAT | 30 | GGGTTGTACTATTGTTTAA | 30 | GGGCGGACGTATAAGACAT | 30 | CCATACATATAGATTGATT |
