# Supplementary figures and images for: LINC00261 and the Adjacent Gene FOXA2 Are Epithelial Markers and Are Suppressed during Lung Cancer Tumorigenesis and Progression
Source: Noncoding RNA. 2018 Dec 28;5(1):2. doi: 10.3390/ncrna5010002 (PMC6468413; doi:10.3390/ncrna5010002)

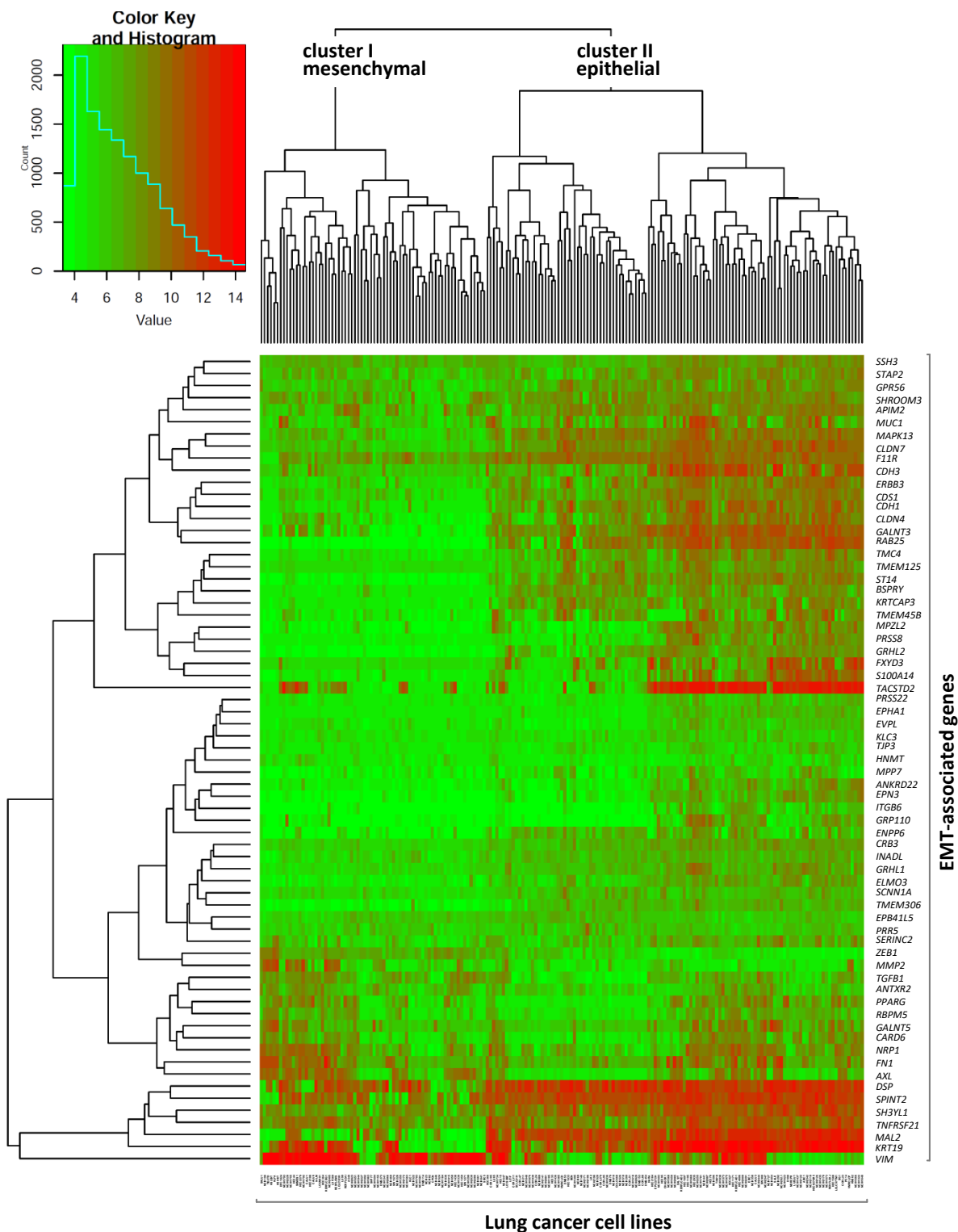

**Figure S2**

Supplement: Supplementary file 1 [file ncrna-05-00002-s001.zip › Figure S2-HighRes.pdf]
